# Trend of transfusion transmitted infections frequency in blood donors: provide a road map for its prevention and control

**DOI:** 10.1186/1479-5876-10-20

**Published:** 2012-01-31

**Authors:** Sobia Attaullah, Sanaullah Khan, Jabbar Khan

**Affiliations:** 1 Department of Zoology, Islamia College Peshawar (A Public Sector University), University Campus, Jamrod Road, Peshawar 25120, Khyber Pakhtunkhwa, Pakistan; 2 Molecular Parasitology and Virology Laboratory, Department of Zoology, Kohat University of Science and Technology, Kohat 26000, Khyber Pakhtunkhwa, Pakistan; 3 Department of Biological Sciences, Gomal University, D.I.Khan, Pakistan

**Keywords:** TTIs, HBV, HCV, HIV, Syphilis, Blood donor

## Abstract

**Background:**

Transfusion transmitted infections create significant burden on health care system. Donor selection is of paramount importance because infected individuals serve as an asymptomatic reservoir and a potential source of transmission.

**Methods:**

A retrospective study was carried out in healthy blood donors in the Lady Reading Hospital Peshawar, Pakistan over a period of three and a half years i.e., from January 2008 to June 2011, to determine the prevalence of HBV, HCV, HIV and syphilis in order to provide information for relevant polices.

**Results:**

Out of 1,27,828 sample of blood donors, recorded mean prevalence for HBs Ag, anti-HCV, anti-HIV and syphilis was 2.68%, 2.46%, 0.06% and 0.43%, respectively, with an increasing trend in frequencies of transfusion transmitted infections (TTIs).

**Conclusions:**

This study reflects that blood transfusion is one of the leading risk factor of spread of the TTIs, which showed the need and importance of the mandatory screening of these infectious markers in blood donations.

## Background

Hepatitis B virus (HBV), hepatitis C virus (HCV), Human immune deficiency virus (HIV) and syphilis are the most important lethal agents in transfusion transmitted infections (TTIs) and it remains a large health care burden globally. The incidence rates across the world are difficult to calculate given the asymptomatic and often latent nature of the disease prior to clinical presentation [1]. Every blood transfusion therefore carries a potential risk for transmissible diseases [1,2].

TTIs are significant contemporary for medicine and society problem in Pakistan, but there exact burden is still unknown due to lack of awareness, poor use of screening tests or their high cost, limited access to a health facility, nonexistence of surveillance system and due to there asymptomatic or have non-specific symptoms. The incidence rates across the world have declined due to the awareness while Pakistan is still in the state of war against these killer infections [3,4].

Blood transfusion is a therapeutic procedure, as there is no genuine substitution. But contaminated blood transfusion can transmit infectious diseases and can be fatal instead of saving life. Safe blood transfusion services are a cornerstone of an effective high quality health care system [5] and require organized infrastructure, properly trained and well-educated staff, availability of expensive equipments and good reagents and continuous supply of electricity. It is however important to mention that TTIs is associated with low viral titer, thus screening of blood donor through molecular mean was believed to be more reliable method for detection [2,6]. Data on the safety of blood transfusion process in Pakistan are scanty and majority of blood banks are not providing safe services according to the recommendation of World Health Organization (WHO) criteria. Poorly organized transfusion network likely contributes significantly in transmission of these serious infectious diseases [7,8]. The dangers of blood transfusions were compounded by poverty as in few blood banks, the screening of the selective blood samples depends upon the recipient's willingness to pay for the cost of the screening. Moreover, 66% of Pakistanis are reside in rural areas with limited access to blood transfusion services [8,9]. Therefore stringent screening of blood donors for TTIs is crucial to ensure safe supply of blood and blood products [1].

Evaluation of data on the prevalence of TTIs among blood donors permits an assessment of the accurate estimate of risk of TTIs which helps in the creation of long-term strategies to improve public health and to prevent spreading of the disease in the local population [1]. The aim of current study was to provide the detail epidemiological analysis of TTIs in blood donors of Peshawar, Khyber Pakhtunkhwa, Pakistan.

## Methods

This study was conducted to evaluate the prevalence of TTIs in healthy blood donors.

### Ethical approval

This study was conducted with the approval of Superintended of the source hospital.

### Study design

It was hospital based retrospective and descriptive study.

### Study location

The data was collected from the blood bank of Lady Reading Hospital (LRH), Postgraduate Medical Institute, Peshawar Khyber Pakhtunkhwa, Pakistan over a period of three and half years. LRH is 1450 bed hospital and has been providing tertiary health care delivery and teaching facilities to population all over the province. Coupled with presence of 0.6 million Afghan refugees and their rush on this hospital, the institution seems over-burdened given its present physical size, administrative human and financial resources. The screening of blood for TTIs is mandatory for blood safety in the source hospital.

### Study population

127828 blood donors of both sexes attended the blood bank during this period which was screened for HBs Ag, anti-HCV, anti-HIV and syphilis.

### Study duration

Records were collected from blood bank of all donors coming to blood bank of LRH during 1^st ^January 2008 to 31^st ^June 2011.

### Inclusion criterion

Selection criteria followed in blood donors is age between 18 to 60 years.

### Exclusion criterion

An exclusion criterion is the pervious history of HBV, HCV and HIV infections. Before screening, all blood donors were subjected to routine physical checkups for exclusion criteria. Apparently unhealthy or malnourished individuals were also refused for blood donations.

### Laboratory test

Blood sample was collected and tested in the blood bank of LRH. HBs Ag, anti-HCV, anti-HIV and Venereal Disease Research Laboratory Test (VDRL) for Syphilis were detected using BEST 2000 ELISA (Biokit, Spain), according to manufacturer's instructions. All seropositive samples were re-tested for the confirmation with the same serological test kit for confirmation.

### Limitation

The study was based on record of blood bank, which did not offer record management of blood donors related to risk factors, thus follow up was not possible in the confirmed positive cases. The same situation exists all across the country due to the heavy work load and dearth of manpower in hospitals.

### Statistical analysis

Data was statistically analysed with statistix9 software. A *p *value less than 0.05 was set as significant level.

## Results

### HBV infection

HBs Ag positive rate for blood donors was found to be 2.68% (3432).

### HCV infections

Anti-HCV prevalence was found to be 2.46% (3147).

### HIV infections

0.06% (77) was found to be positive for anti-HIV.

### VDRL infection

VDRL was positive in 0.43% (544) blood donors.

**Figure 1:**
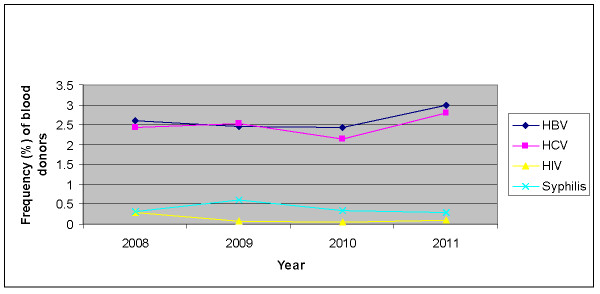
**Trend in the prevelence of HBV, HVV, HIV and Syphilis in blood donors from 2008 to 2011**.

Table 1 showed the epidemiological rate and statistical figure for TTIs in blood donors during the whole study period. Majority of donors were male (male = 127780 and female = 48). None of the female blood donor was found positive to TTIs. Over all, HBV rate is high when compared to the prevalence HIV and syphilis. This study indicated downward trend in the HCV, HIV and syphilis while significant increase was noted in figure of HBV and HCV in 2011(Figure 1).

**Table 1:** Prevalence of TTIs in Blood Donors from January 2008 to June 2011

Year	Month	HBV	HCV	HIV	Syphilis	Total
2008	January	70	67	0	5	2746
	
	February	99	56	0	11	2928
	
	March	105	75	1	4	3500
	
	April	73	74	1	6	3246
	
	May	97	89	1	5	3534
	
	June	65	66	1	4	3229
	
	July	75	84	0	4	3557
	
	August	78	85	1	5	3283
	
	September	96	91	2	9	3212
	
	October	83	94	2	21	3449
	
	November	94	96	2	34	3096
	
	December	82	71	0	18	3220
	
	**Total**	**1017**	**948**	**11**	**126**	**39000**

2009	January	75	120	0	11	3160
	
	February	111	79	2	16	2914
	
	March	69	110	1	17	3157
	
	April	98	98	5	35	3485
	
	May	84	84	1	24	3388
	
	June	70	70	3	18	3002
	
	July	82	75	2	22	3535
	
	August	74	68	1	19	3537
	
	September	52	46	2	23	2848
	
	October	83	77	2	30	3525
	
	November	60	61	9	6	2743
	
	December	86	83	4	9	3293
	
	**Total**	**944**	**971**	**32**	**230**	**38587**

2010	January	94	70	0	8	3120
	
	February	72	84	0	11	3052
	
	March	99	102	1	20	3674
	
	April	106	93	1	22	3683
	
	May	98	88	4	14	3994
	
	June	63	50	2	14	3220
	
	July	73	59	2	7	3355
	
	August	65	64	3	15	3078
	
	September	91	81	2	11	3568
	
	October	74	57	5	7	3277
	
	November	67	69	0	7	3306
	
	December	77	51	4	7	3149
	
	**Total**	**979**	**868**	**24**	**143**	**40476**

2011	January	67	45	2	3	1123
	
	February	68	62	0	6	1815
	
	March	86	50	3	3	1005
	
	April	95	73	2	13	1811
	
	May	77	61	0	7	2110
	
	June	99	69	3	13	1901
	
	**Total**	**492**	**360**	**10**	**45**	**9765**

**Grand total** **(*P *value)**		**3432** **(0.0060)**	**3147** **(0.0059)**	**77** **(0.0050)**	**544** **(0.0051)**	**127828**

## Discussion

Globally mass of evidence supported that blood transfusion is an efficient route of TTIs transmission [3]. According to the WHO, in Pakistan about 1.5 million units of blood products transfused annually [8]. 15% of blood is professional donation, 75% are replacement (hidden payment) donation while only 10% are voluntary unpaid donations [10]. Blood transfusion departments or blood banks not only screen the blood but also give clue about rate of prevalence of TTIs [3]. Ali and colleagues [7] reported that overall HBs Ag and HCV prevalence was higher in the non blood donor population (3.8% weighted average, range 1.4-11%; 5.4% weighted average, range 2.1-31.9%) than blood donor population (2.3% weighted average, range 1.4-8.4%; 2.8% weighted average, range 0.5-20.7%). It requires highlighting the strategies to identify, recruit and retain donors from low-risk populations. The epidemiological characteristics and risk factors for transmission vary across country [11].

Hakim et al., [12] from over 350 reports, papers and presentations estimated the combined prevalence of HBV and HCV in various parts of country at 8-10% and a substantial decline in HBV has been observed due to mass scale immunization program against HBV infection while prevalence of HCV has risen due to unavailability of vaccine. Farooqi et al., [13] documented 1,66,189 blood donors (from year 2001 to 2006) from the same hospital and reported 2.54% mean prevalence for HBV and 3.21% for HCV. The findings of the current study was in line with the reports of high prevalence of HBs Ag and comparatively low prevalence of HCV [12,13].

Limited surveillance data exist for HIV and syphilis in Pakistan. In 1992 prevalence of HIV in blood donors of same hospital was estimated to be 0%, 0.0086% (2/23,278) in 2002 [14] while current study reported the figure of 0.06%. it showed the rising burden of HIV and demonstrated a clear need for an integrated cross-disciplinary and regional approach to the emerging threat of HIV. Our country still has a window of opportunity to act decisively to prevent its spread. Important issue is to not neglect the rising rates of HIV in neighboring Asian nations as 0.35% in India [15]. Syphilis seemed to have been controlled over the years, but now re-emerged as a major public health problem in many communities [4]. A VDRL test was found to be positive in 0.5% blood donors in Lahore [5]. Current result (0.43%) showed high prevalence in Peshawar blood donors.

Investigation of the prevalence TTIs is often conducted in blood donor population because of convenience and access to a large sample size [7]. They are generally considered a healthier segment of the community and have been used as a surrogate marker for the prevalence of infection in the population at large [9,16]. In Pakistan majority of blood donors are first time blood donors and can be considered true reflector of infection among the community [3].

According to other authors [7,17], blood donors may not be considered the representative of general population as the prevalence rate may underestimate or overestimate due to their different characteristics. Prevalence source may underestimate the real prevalence due to certain limitations as most of them included men, young or middle aged people. So the prevalence rate was missed in other age groups as well as in female's population. Prevalence rate may be an overestimate if professional blood donors were injecting drug users who sold blood for money and found highly engaged in unprotected sex with commercial partners [7]. Other important issue is that the main source of replacement in our region includes donors recruited from among a patient's family or acquaintances who hide their health conditions from their relatives. Thus selection of donor and their proper screening are key factors to ensure safe transfusion [9].

One probable reason for high prevalence rate in this study may be inclusion of professional blood donors, but still it can not eliminate the threat of disease spreading to other populations. On other hand if major blood donors were volunteers, than these figures is alarming sending a clear warning the health care and policy makers about the control of this monster. However, majority of blood donor's data set in Pakistan including current study cannot evaluate the exact situation as potential donors with a high-risk profile (like history of jaundice, injection drug use, multiple sexual partners, etc.), has screened out only by questionnaires. The Department of Health is advised to conduct sentinel surveys for blood banks to contribute significantly for the identification and monitoring of high-risk blood donors.

Government of Pakistan has introduced a national blood policy of proper screening of blood before recommending for transfusion as late as 2003 [9]. In Brazil, screening test among blood donors was mandatory in 1993. That is one of the significant factors of low magnitude of hepatitis in all studied population of Brazil as compared to Pakistan (1.42% to 1.7% vs. 4.95% ± 3.5% in the general population, 0.9% vs. 3.78% ± 0.41 in blood donors and 23.8% to 52.0% vs. 23.7% to 68.0% in haemodialysis patients) [9,18]. The risk of hepatitis transmission through blood transfusion is considered to be high in Pakistani population due to a lack of appropriate screening of blood in past. Late vaccination also resulted in spreading of HBV. Several studies confirmed the prevalence of hepatitis among people with a history of blood transfusion before the advent of blood screening procedures [2,6].

### Guide line

Blood transfusion is an important preventable modality of spread of TTIs. It is a time of urgent and concrete measures to eliminate the transfusions from paid donors and to improve the safety of the blood supply. To provide proper transfusion facilities to underdeveloped areas requires economic growth. These conditions can be overcome by development of a fair and organized system of blood screening and transfusion [9]. This debate should count sufficient to sensitize the policy maker about the gravity of situation.

## Conclusions

Despite stringent donor screening and testing practices, safe blood free from TTIs remains an elusive goal because the threat of TTIs agents entering the blood supply is not static. This finding showed growing evidence in the burden of TTIs in blood donors. The field of transfusion medicine has encountered huge precautions in providing safe blood. Prevention of spread of TTIs should be the main goal at the current time, therefore number of indicators, both quantitative and qualitative, for monitoring the implementation strategies for recruitment and retention of safe regular donors is the need of the time.

## Competing interests

The authors declare that they have no competing interests.

## Authors' contributions

SA and SK designed, searched data and literature and gave a critical view of manuscript writing. JK gave critical view of manuscript writing. All the authors' read and approved the final manuscript.

## References

[B1] Bhawani Y Rao PR Sudhakar V Seroprevalence of transfusion transmissible infections among blood donors in a tertiary care hospital of Andhra Pradesh Biol Med 2010 2 4 45 48

[B2] Khan S Attaullah S Ayaz S Khan SN Shams S Ali I Bilal M Siraj S Molecular epidemiology of HCV among the health care workers of Khyber Pakhtunkhwa Virol J 2011 8 1 105 10.1186/1743-422X-8-105 21385397 PMC3060846

[B3] Khan ZT Asim S Tariq Z Ehsan MA Malik RA Ashfaq B Hayat Prevelence of transfusion transmitted infectious in healthy blood donors in Rawalpindi District, Pakistan: a five year survey Inter J of Pathology 2007 5 1 21 25

[B4] Shah SA Usman G Ghazi A Kristensen S Sathiakumar N Memon MA John R Vermund SH Prevalence of syphilis among antenatal clinic attendees in Karachi: imperative to begin universal screening in Pakistan JPMA 2011 61 993 PMC3574871 22356034

[B5] Manzoor I Hashmi N Daud S Seroprevalence of transfusion transmissible infections (TTIS) in blood donors Biomedica 2009 25 154 158

[B6] Attaullah S Khan S Ayaz S Khan SN Ali I Hoti N Siraj S Prevalence of HBV and HBV Vaccination Coverage in Health Care Workers of Tertiary Hospitals of Peshawar, Pakistan Virol J 2011 8 1 275 10.1186/1743-422X-8-275 21645287 PMC3121707

[B7] Ali SA Donahue RMJ Qureshi H Vermund SH Hepatitis B and Hepatitis C in Pakistan: prevelence and risk factors Int J Infec Dis 2009 13 1 9 19 10.1016/j.ijid.2008.06.019 PMC2651958 18835208

[B8] Kassi M Khanani MR Khan IA Ali SH Safe blood transfusion practices in blood banks of Karachi, Pakistan Transfusion Med 2011 21 57 62 10.1111/j.1365-3148.2010.01042.x 21029219

[B9] Waheed Y Shafi T Safi SZ Qadri I Hepatitis C virus in Pakistan: a systematic review of prevalence, genotypes and risk factors World J Gastroenterol 2009 15 45 5647 5653 10.3748/wjg.15.5647 19960560 PMC2789216

[B10] National Blood Policy and Strategic Framework 2008-2012 For Blood Transfusion Services in Pakistan. National AIDS Control Program Ministry of Health, Government of Pakistan http://www.nbtp.gov.pk/Docs/National%20Blood%20Policy

[B11] Khan EA Khokhar N Malik GJ Seroprevalence of syphilis in asymptomatic adults seeking employment abroad Rawal Med J 2004 29 2 65 67

[B12] Hakim ST Kasmi SU Bagasara O Seroprevelence B and C Genotypes among young apparently healthy female of Karachi-Pakistan Libyan J Med 2008 3 66 70 10.4176/071123 21499460 PMC3074282

[B13] Farooqi JI Farooqi RJ Khan N Mussarat Frequency of hepatitis B and C in selected groups of population in NWFP, Pakistan JPMI 2007 21 3 165 167

[B14] Khan Z Raziq F Aslam N Prevalence of HIV in blood donors in NWFP JPMI 2002 16 2 187 189

[B15] Bhattacharya P Chandra PK Datta S Banerjee A Chakravarty R Significant increase in HBV, HCV, HIV and syphilis infections among blood donors in West Bengal, Eastern India 2004-2005: exploratory screening reveals high frequency of HBV infection World J Gastroenterol 2007 13 27 3730 3733 17659734 10.3748/wjg.v13.i27.3730 PMC4250646

[B16] Meena M Jindal T Hazarika A Prevalence of hepatitis B virus and hepatitis C virus among blood donors at a tertiary care hospital in India: a five-year study Transfusion 2011 51 198 202 10.1111/j.1537-2995.2010.02801.x 20663107

[B17] Jafri W Jafri N Yakoob J Islam M Tirmizi FA Jafar T Akhtar S Hamid S Shah HA Nazmi SQ Hepatitis B and C: prevalence and risk factors associated with seropositivity among children in Karachi Pakistan BMC Infect Dis 2006 6 101 10.1186/1471-2334-6-101 16792819 PMC1539007

[B18] Resende VL Abreu MH Paiva SM Teixeira R Pordeus SA Factors associated with seroprevalence of hepatitis C among dentists at a large Brazilian city Virol J 2009 6 228 10.1186/1743-422X-6-228 20030849 PMC2806292

